# Expression patterns of STAT3, ERK and estrogen-receptor α are associated with development and histologic severity of hepatic steatosis: a retrospective study

**DOI:** 10.1186/s13000-018-0698-8

**Published:** 2018-04-03

**Authors:** Euno Choi, Won Kim, Sae Kyung Joo, Sunyoung Park, Jeong Hwan Park, Yun Kyung Kang, So-Young Jin, Mee Soo Chang

**Affiliations:** 1 Department of Pathology, Seoul National University Boramae Hospital, Seoul National University College of Medicine, 20 Boramae-ro 5-gil, Dongjak-gu, Seoul, 07061 Korea; 2 Department of Internal Medicine, Seoul National University Boramae Hospital, Seoul National University College of Medicine, 20 Boramae-ro 5-gil, Dongjak-gu, Seoul, Korea; 3 Department of Pathology, Seoul Paik Hospital, Inje University College of Medicine, Mareunnae-ro 9, Jung-gu, Seoul, Korea; 4 Department of Pathology, Soon Chun Hyang University Hospital, 59 daesagwan-ro, Yongsan-gu, Seoul, Korea

**Keywords:** Hepatic steatosis, Non-alcoholic, Alcoholic, mTOR, pSTAT3, pERK, Estrogen-receptor α

## Abstract

**Background:**

Hepatic steatosis renders hepatocytes vulnerable to injury, resulting in the progression of preexisting liver disease. Previous animal and cell culture studies implicated mammalian target of rapamycin (mTOR), signal transducer and activator of transcription-3 (STAT3), extracellular signal-regulated kinase (ERK) and estrogen-receptor α in the pathogenesis of hepatic steatosis and disease progression. However, to date there have been few studies performed using human liver tissue to study hepatic steatosis. We examined the expression patterns of mTOR, STAT3, ERK and estrogen-receptor α in liver tissues from patients diagnosed with hepatic steatosis.

**Methods:**

We reviewed the clinical and histomorphological features of 29 patients diagnosed with hepatic steatosis: 18 with non-alcoholic fatty liver disease (NAFLD), 11 with alcoholic fatty acid disease (AFLD), and a control group (16 biliary cysts and 22 hepatolithiasis). Immunohistochemistry was performed on liver tissue using an automated immunostainer. The histologic severity of hepatic steatosis was evaluated by assessing four key histomorphologic parameters common to NAFLD and AFLD: steatosis, lobular inflammation, ballooning degeneration and fibrosis.

**Results:**

mTOR, phosphorylated STAT3, phosphorylated pERK, estrogen-receptor α were found to be more frequently expressed in the hepatic steatosis group than in the control group. Specifically, mTOR was expressed in 78% of hepatocytes, and ERK in 100% of hepatic stellate cells, respectively, in patients with NAFLD. Interestingly, estrogen-receptor α was diffusely expressed in hepatocytes in all NALFD cases. Phosphorylated (active) STAT3 was expressed in 73% of hepatocytes and 45% of hepatic stellate cells in patients with AFLD, and phosphorylated (active) ERK was expressed in hepatic stellate cells in all AFLD cases. Estrogen-receptor α was expressed in all AFLD cases (focally in 64% of AFLD cases, and diffusely in 36%). Phosphorylated STAT3 expression in hepatocytes and hepatic stellate cells correlated with severe lobular inflammation, severe ballooning degeneration and advanced fibrosis, whereas diffusely expressed estrogen-receptor α correlated with a mild stage of fibrosis.

**Conclusions:**

Our data indicate ERK activation and estrogen-receptor α may be relevant in the development of hepatic steatosis. However, diffuse expression of estrogen-receptor α would appear to impede disease progression, including hepatic fibrosis. Finally, phosphorylated STAT3 may also contribute to disease progression.

**Electronic supplementary material:**

The online version of this article (10.1186/s13000-018-0698-8) contains supplementary material, which is available to authorized users.

## Background

Hepatic steatosis is a frequent histological finding in liver biopsy specimens. The causes of hepatic steatosis include obesity, excessive alcohol intake, chronic autoimmune diseases, Wilson’s disease, hepatitis C virus and certain pharmacological drugs [1]. Increasing evidence indicates that hepatic steatosis enhances hepatocellular susceptibility to additional injuries, eventually leading to hepatic fibrosis [2]. The molecular factors contributing to hepatic steatosis, hepatocellular damage, and hepatic fibrosis have been studied, but their action mechanisms are still not fully resolved.

The mammalian target of rapamycin (mTOR) controls lipid biosynthesis via various effector molecules, such as sterol regulatory element-binding protein like-1c (SREBP-1c) [3] which engages in the development of hepatic steatosis [4]. mTOR, signal transducer and activator of transcription-3 (STAT3), and extracellular signal-regulated kinase (ERK) all belong to downstream mediators of leptin signaling [5–7], and it has been shown that leptin plays a crucial role in hepatic lipid regulation [8]. In cell experiments, signal transducer and activator of transcription (STAT3) has been shown to be required for IL-6-mediated activation of hepatic stellate cells, eventually resulting in hepatic fibrosis [9]. Also, the kinases, including extracellular signal-regulated kinase (ERK), are activated early in liver injury, and then led to hepatic fibrosis of rats [10]. In addition, estrogen is a steroid hormone that preserves hepatic lipid hemostasis by acting via estrogen-receptor α [11, 12]. Animal and cell culture studies suggest estrogen prevents hepatic fibrosis by blocking lipid peroxidation and production of reactive oxygen species [13].

To date, the cellular localization and expression patterns for leptin signaling proteins and estrogen-receptor α have not been collectively determined in liver tissue, and specifically in hepatocytes and hepatic stellate cells. Hepatic stellate cells line the perisinusoidal space, and are located between hepatocytes and the sinusoidal endothelium [14]. When activated by liver injury, these cells newly express α-smooth muscle actin and transdifferentiate into myofibroblast-like cells, which form the extracellular matrix that leads to hepatic fibrosis [14–16].

Non-alcoholic fatty liver disease (NAFLD) and alcoholic fatty liver disease (AFLD) are two common causes of chronic liver disease worldwide [17]. Although managed differently [18], NAFLD and AFLD possess similar histomorphologic features (e.g., macrovesicular steatosis, lobular inflammation, and hepatocyte ballooning degeneration) [19–21] and a similar course of disease progression (simple steatosis followed by steatohepatitis, fibrosis, and micronodular cirrhosis) [21–23]. They have shared and disease-specific mechanisms of lipid accumulation, and their pathogenesis includes oxidative stress, iron deposition, overexpression of cytochrome P450E1, and involvement of endotoxins and tumor necrosis factor α [19–21]. Oxidative stress induces expression of lipid metabolism-associated transcription factors (e.g., SREBP-1c) that regulate de novo fatty acid synthesis [4]. Disease-specific mechanisms include augmented lipolysis in adipose tissue and consequent increases in circulating free fatty acid levels in NAFLD, but not in AFLD [20]. The major substrate of cytochrome P450E1 is excessive free fatty acids in NAFLD and ethanol in AFLD [4, 19, 24].

Here, we evaluated the clinical and histomorphologic features and expression patterns of mTOR, phosphorylated STAT3 (pSTAT3), phosphorylated ERK (pERK) and estrogen-receptor α in hepatocytes and hepatic stellate cells in liver tissue from patients with hepatic steatosis, along with a control group (biliary cysts and hepatolithiasis). Our goal was to determine the roles these proteins might play in the development of hepatic steatosis.

## Methods

### Patients and liver tissues

This retrospective study comprised 29 patients with hepatic steatosis; 18 diagnosed with NAFLD and 11 diagnosed with AFLD. Patients were selected from the electronic database of the Department of Pathology, Seoul National University Boramae Hospital. Information in the database was obtained from needle biopsies of liver tissue during 2005–2011. Diagnoses were based on clinicopathological correlations, the results of a serologic test of liver function, causes of the liver dysfunction, a history of alcohol intake, and histomorphologic evaluation of liver tissue biopsies [25, 26]. Inclusion criterion for liver biopsies was a tissue specimen > 1 cm in length without fragmentation or > 10 portal tracts with macrovesicular steatosis in > 5% of hepatocytes.

Results of a serologic test of liver function were regarded as abnormal if aspartate transaminase (AST) or alanine transaminase (ALT) levels were each > 40 U/L and total bilirubin levels were > 1.2 mg/dL. Body mass index (BMI) was determined as body weight in kilograms divided by height in meters squared. According to the revised World Health Organization criteria for obesity in the Asia Pacific region, BMIs > 25, 23–24.9, 18.5–22.9, and < 18.5 correspond to obesity, overweight, normal heathy weight, and underweight, respectively [27]. Alcoholic fatty liver disease was considered if alcohol consumption was excessive (> 30 g/day for men and > 20 g/day for women) as per each patient’s self-reporting and interviews with family members.

To better understand protein expression patterns in non-neoplastic hepatocytes, we used a control group composed of surgically resected liver tissue from 16 cases of biliary cysts and 22 cases of hepatolithiasis (without steatosis, fibrosis or dysplasia).

### Histomorphologic assessment

Formalin-fixed, paraffin-embedded (FFPE) tissue samples were microsectioned and stained with both hematoxylin and eosin and Masson’s trichrome stain. Diagnoses of NAFLD and AFLD were based on clinical features and pathological criteria [23, 25, 26]. Common histomorphologic features of NAFLD and AFLD include the four core features of steatosis, lobular inflammation, ballooning degeneration, and fibrosis, all of which were assessed using the Non-alcoholic Steatohepatitis Clinical Research Network (NASH CRN) NAFLD activity score (NAS) system [26] and the descriptive system proposed for alcoholic liver disease [23]. NAFLD was also sub-classified as significant or mild using the steatosis, activity, and fibrosis (SAF) scoring system [28]. The SAF and NASH CRN NAS systems apply similar criteria when determining grades and scores for steatosis, activity and fibrosis. The main difference is the evaluation criteria of ballooning degeneration; SAF system puts the size and number of the enlarged cells into consideration while the NASH CRN system considers only the number of enlarged cells [29].

### Immunohistochemistry

Immunohistochemistry was performed using an automated Ventana Benchmark XT immunostainer (Ventana BenchMark XT; Ventana Medical Systems Inc., Tucson, AZ, USA) according to the manufacturer’s protocol. Briefly, 3-μm-thick tissue sections were placed on electrostatic charged glass slides, deparaffinized, and subjected to antigen retrieval. The antigen was detected using ultraView Universal DAB Detection Kit (Ventana Medical Systems Inc.). For double staining, an ultraView Universal Alkaline Phosphatase Red Detection Kit (Ventana Medical Systems Inc.) was used. The following primary antibodies were used at the following dilutions: mTOR (49F9, 1:50; Cell Signaling, Danvers, MA, USA), pSTAT3 (1:50; Cell Signaling), pERK1/2 (44/42 MAPK, Thr 202/Tyr 204) (E10, 1:50; Cell Signaling), estrogen-receptor α (SP1, ready to use; Ventana Medical Systems Inc.), and α-smooth muscle actin (1A4, 1:500; Labvision, Fremont, CA, USA). For negative controls, each antibody was replaced with phosphate-buffered saline.

For mTOR and the phosphorylated (i.e., active) forms of STAT3 and ERK, nuclear staining was evaluated and considered positive when ≥10% of hepatocytes, or any of the hepatic stellate cells, were stained. For estrogen-receptor α expression, the area of positively stained hepatic nuclei was categorized as 1 (< 10%), 2 (10–50%), or 3 (> 50%), and staining intensity was graded as 1 (weak), 2 (moderate), or 3 (strong). A total score (1 to 9) was calculated by multiplying area and intensity scores. Estrogen-receptor α expression was also defined as negative, focal, or diffuse (scores of 1, 2–4, and 6–9, respectively). α-smooth muscle actin was used as a marker for hepatic stellate cells [13, 14].

### Statistical analysis

Statistical differences were analyzed using the chi-square test or Fisher’s exact test (2-sided) for categorical variables, and the Mann-Whitney U test or analysis of variance for continuous variables. *P* values <0.05 were considered statistically significant. Statistical analyses were performed using SPSS Statistics version 20.0 (IBM Inc., Armonk, NY, USA).

## Results

### Clinical and histomorphologic features of hepatic steatosis

**Fig. 1:**
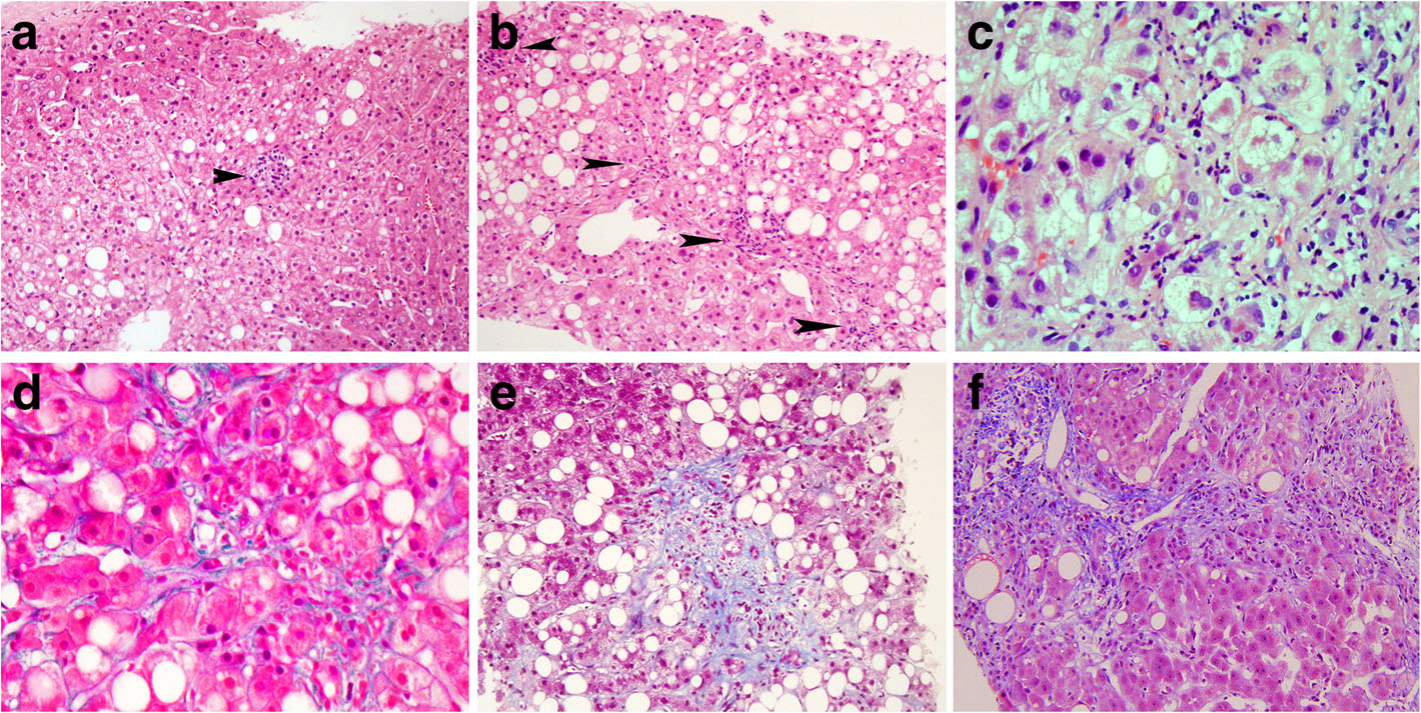
Representative histomorphologic features of hepatic steatosis. (**a**) Lobular inflammation, with a single focus of inflammatory cells (arrow) (H&E). (**b**) Lobular inflammation, with four foci of inflammatory cells (arrows) (H&E). (**c**) Ballooning degeneration, with many enlarged hepatocytes including at least one twice the size of a normal cell (H&E). (**d**) Perisinusoidal fibrosis (Masson’s trichrome). (**e**) Periportal fibrosis (Masson’s trichrome). (**f**) Septal fibrosis (Masson’s trichrome)

Clinical and histomorphologic characteristics of patients with hepatic steatosis and a control group (biliary cysts and hepatolithiasis cases without steatosis, fibrosis or dysplasia) are summarized in Table 1. Most NAFLD patients were obese, whereas 55% of AFLD patients were of normal weight (*P* = 0.001). An AST/ALT ratio ≤ 2 was more often observed in the NAFLD group than the AFLD group, but the difference was not statistically significant. The NAFLD group showed milder histomorphologic features in lobular inflammation, ballooning degeneration and fibrosis than the AFLD group (*P* < 0.05, respectively). Representative histomorphologic features are shown in Fig. 1.

**Table 1:** Clinicopathologic and histomorphologic features in hepatic steatosis patients

	NAFLD (*n* = 18)	AFLD (*n* = 11)	Control (*n* = 38)
Clinical Features			
Age, mean (range) years	23 ± 6.4 (11–36)^a^	41 ± 9.5 (23–59)^a^	61 ± 12.5 (34–89)^a^
Sex			
male	15 (83%)	9 (82%)	11 (29%)
female	3 (17%)	2 (18%)	27 (71%)
BMI			
underweight	0	1 (9%)	2 (5%)
normal	3 (17%)	6 (55%)	13 (34%)
overweight	0	2 (18%)	15 (39%)
obese	15 (83%)	2 (18%)	8 (21%)
Liver Biochemistry			
AST/ALT ratio			
≤2	14 (78%)	6 (55%)	–
> 2	4 (22%)	5 (45%)	–
AST, median (mean, range) U/L	79 (67, 11~ 192)	67 (105, 21~ 152)	22 (35, 12~ 249)
ALT, median (mean, range) U/L	84 (124, 11~ 384)	44 (35, 12~ 204)	17 (31, 7~ 252)
TB, median (mean, range) mg/dL	0.8 (0.9, 0.5~ 12.4)	14 (9, 0.7~ 19.8)	0.7 (0.8, 0.3~ 3.5)
Histomorphological Features			
Steatosis			None
5–33%	4 (22%)	2 (18%)	
> 33–66%	5 (28%)	5 (45%)	
> 66%	9 (50%)	4 (36%)	
Lobular inflammation (inflammatory foci/200× field)			None
< 2	16 (89%)	6 (55%)	
2–4	2 (11%)	3 (27%)	
> 4	0	2 (18%)	
Ballooning degeneration			None
none	9 (50%)	1 (9%)	
few	8 (44%)	5 (45%)	
many	1 (6%)	5 (45%)	
Fibrosis			None
none	6 (33%)	1 (9%)	
perisinusoidal or peripota1	11 (61%)	1 (9%)	
Perisinusoidal & potal/periportal	1 (6%)	1 (9%)	
bridging fibrosis	0	3 (27%)	
cirrhosis	0	5 (45%)	

*NAFLD* non-alcoholic fatty liver disease

*AFLD* alcoholic fatty liver disease

*Control,* 16 cases of biliary cyst and 22 cases of hepatolithiasis

^a^mean ± standard deviation

*AST* aspartate transaminase

*ALT* alanine transaminase

*TB* total bilirubin

## Protein expression patterns in hepatic steatosis

**Fig. 2:**
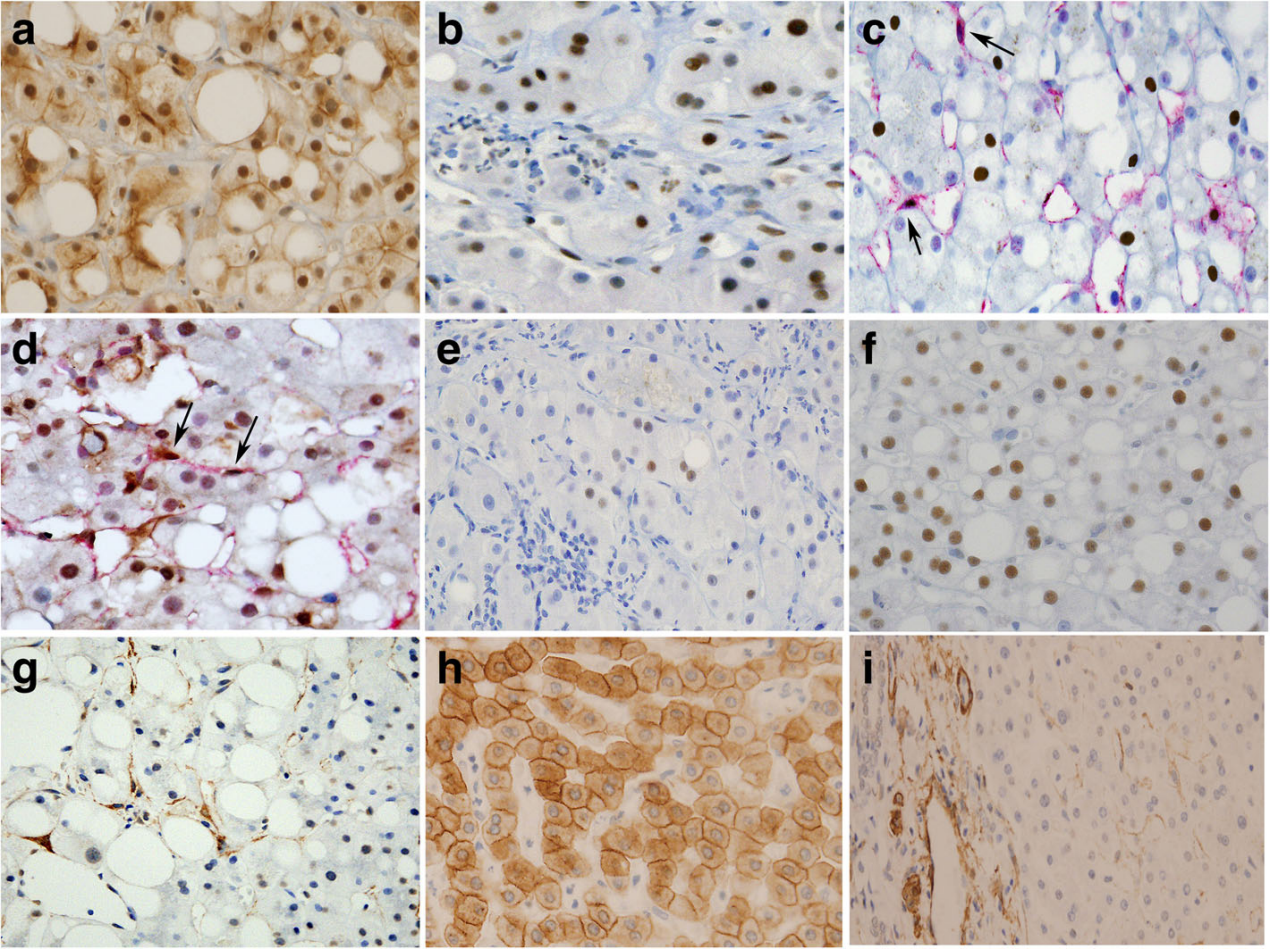
Representative immunohistochemical features in hepatic steatosis (**a-g**) and the control group (**h-i**). (**a)** Nuclear and/or weak cytoplasmic and membranous expression of mammalian target of rapamycin (mTOR). (**b**) Nuclear staining of phosphorylated signal transducer and activator of transcription-3 (pSTAT3) in hepatocytes. (**c**) Double staining of pSTAT3 (nucleus) and α-smooth muscle actin (cytoplasm, red color) in hepatic stellate cells (arrows). (**d**) Double staining of extracellular signal-regulated kinase (pERK) (nucleus) and α-smooth muscle actin (cytoplasm, red color) in hepatic stellate cells (arrows). (**e**) Focal expression of estrogen-receptor α in hepatocyte nuclei. **(f**) Diffuse expression of estrogen-receptor α. (**g**) α-smooth muscle actin-positive cytoplasm in hepatic stellate cells. (**h**) Cytoplasmic and membranous staining of mTOR in non-neoplastic hepatocytes in a control sample. (**i**) Inconspicuous staining of α-smooth muscle actin (inactive hepatic stellate cells; center of figure) in a control sample, while strong positive staining in vessel walls as an internal positive control (left side of figure)

The hepatic steatosis group frequently showed more positive expression of proteins than the control group (*P* < 0.05 for the most proteins). The only exception to this trend was pSTAT3 in hepatic stellate cells which showed marginal expression differences (*P* = 0.055) (Table 2). In detail, hepatic mTOR was expressed in 78% of NAFLD cases. Of interest, estrogen-receptor α expression was diffusely positive in all NAFLD cases. NAFLD cases were also characterized by low (hepatocytes, 17%) or no (hepatic stellate cells) pSTAT3 expression, and low (hepatocytes, 22%) or predominant (hepatic stellate cells, 100%) pERK expression. Notably, in AFLD, pSTAT3 was often observed in both hepatocytes (73%) and hepatic stellate cells (45%). All AFLD cases had estrogen-receptor α-positive hepatocytes, with focal positivity in 64% of cases and diffuse positivity in 36%. Estrogen-receptor α was not sufficiently expressed to be considered positive by immunohistochemical evaluation in the non-neoplastic hepatocytes of the control group (biliary cyst and hepatolithiasis cases without hepatic steatosis, fibrosis or dysplasia). Representative immunohistochemical features can be seen in Fig. 2. We observed non-specific cytoplasmic or membranous staining of mTOR in non-neoplastic hepatocytes in all hepatic steatosis and control cases. Hence, only mTOR staining in the nucleus was evaluated.

**Table 2:** Comparison of protein expression features in hepatic steatosis patients and the control group

	Total hepatic steatosis	NAFLD	AFLD	Control	^*^P value
	(*N* = 29)	(*n* = 18)	(*n* = 11)	(*N* = 38)	
Protein expression in hepatocytes					
mTOR, positive	16 (55%)	14 (78%)	2 (18%)	1 (3%)	< 0.001
pSTAT3, positive	11 (38%)	3 (17%)	8 (73%)	4 (11%)	0.007
pERK, positive	8 (28%)	4 (22%)	4 (36%)	1 (3%)	0.003
Estrogen-receptor α, positive	29 (100%)	18 (100%)	11 (100%)	0	< 0.001
Focal positive	7 (24%)	0	7 (64%)	0	
Diffuse positive	22 (76%)	18 (100%)	4 (36%)	0	
Protein expression in hepatic stellate cells					
pSTAT3, positive	5 (17%)	0	5 (45%)	2 (5%)	0.055
pERK, positive	29 (100%)	18 (100%)	11 (100%)	3 (8%)	< 0.001

*NAFLD* non-alcoholic fatty liver disease

*AFLD* alcoholic fatty liver disease

*Control,* non-neoplastic hepatosytes from 16 cases of biliary cyst and 22 cases of hepatolithiasis

^*^ *P* value between total hepatic steatosis group (*N* = 29) and the control group (*N* = 38)

### Relationship between protein expression and histologic severity in hepatic steatosis

Expression of pSTAT3 in hepatocytes and hepatic stellate cells significantly correlated with severe histologic features (e.g., severe lobular inflammation, severe ballooning inflammation, and advanced stage of fibrosis; *P* < 0.05, respectively) (Table 3). In contrast, diffuse nuclear expression of estrogen-receptor α in hepatocytes correlated with mild histologic features (*P* < 0.05). There was no statistical relationship between pERK expression and histologic severity, although there was a near significant relationship (*P* = 0.083) between pERK expression and lobular inflammation in hepatic stellate cells.

**Table 3:** Protein expressions related with histologic severity of hepatic steatosis

	pSTAT3 in hepatocytes			Estrogen-receptor α in hepatocytes			pSTAT3 in hepatic stellate cells		
	negative	positive	*P* value	focal	diffuse	*P* value	negative	positive	*P* value
Lobular inflammation (inflammatory foci/ 200× field)			0.028			0.022			0.016
< 2	16 (55%)	5 (17%)		3 (10%)	18 (62%)		20 (69%)	1 (3%)	
2–4	2 (7%)	4 (14%)		2 (7%)	4 (14%)		3 (10%)	3 (10%)	
> 4	0	2 (7%)		2 (7%)	0		1 (3%)	1 (3%)	
Ballooning degeneration			0.010			0.001			0.033
none	10 (34%)	1 (3%)		0	11 (38%)		11 (38%)	0	
few	7 (24%)	5 (17%)		2 (7%)	10 (34%)		10 (34%)	2 (7%)	
many	1 (3%)	5 (17%)		5 (17%)	1 (3%)		3 (10%)	3 (10%)	
Fibrosis			0.001			0.000			0.000
absent	11 (38%)	0		0	11 (38%)		11 (38%)	0	
mild^a^	6 (231%)	4 (14%)		0	10 (34%)		10 (34%)	0	
advanced^b^	1 (3%)	7 (24%)		7 (24%)	1 (3%)		3 (10%)	5 (17%)	

^a^perisinusoidal, portal &/or periprotal fibrosis

^b^bridging fibrosis or cirrhosis

Using the NASH CRN and SAF histologic scoring systems, we classified NAFLD as mild, severe, or significant. Ballooning degeneration and lobular inflammation were significantly increased in severity in patients with either severe or significant NAFLD when compared to those with mild NAFLD (*P* < 0.05, respectively) (Additional file 1). However, it should be noted that there was no difference in protein expression between the severe/significant subgroup and the mild subgroup.

## Discussion

The present study identifies pSTAT3 as a potential marker of histologic severity in hepatic steatosis. pSTAT3 may be required in disease progression of hepatic steatosis. Our data showed that pSTAT3 expression in hepatocytes and hepatic stellate cells correlated significantly with severe lobular inflammation, severe ballooning degeneration, and advanced fibrosis. These pSTAT3 results are in agreement with previous animal and cell culture studies in which interleukin-6 promoted liver inflammation by activating hepatic STAT3 [30]. To the best of our knowledge, this is the first report of pSTAT3 expression in the liver tissue of hepatic steatosis patients.

Previous studies also found that STAT3 activation in hepatocytes and hepatic stellate cells lead to hepatic fibrosis [31–35]. Here, STAT3 mediated the effects of leptin via collagen gene activation in a liver fibrogenesis mouse model [31, 32]. STAT3 may promote hepatic fibrosis [36, 37] through the upregulation of tissue inhibitor of metalloproteinases-1 [33, 34] and transforming factor-ß expression [37]. On the other hand, repression of STAT3 expression was found to exacerbate liver inflammation in interleukin-10-deficient mice [38] and accelerate hepatic fibrosis during cholestasis [39]. Further study will be required to determine if there might be condition-specific feedback loops that enhance or inhibit STAT3 function.

In hepatic steatosis with severe or advanced histomorphologic features, we found estrogen-receptor α expression was focal rather than diffuse. Our data suggest that diffuse expression of estrogen-receptor α may actually have a protective effect against disease progression, potentially by serving as a hepatic receptor for estradiol [40], which inhibits the generation of reactive oxygen species and suppresses hepatic stellate cell activation, resulting in reduced proliferation and collagen production [13, 41]. Protective effects of estrogen in patients with hepatic fibrosis have been reported by others [41]. Moreover, an estradiol study where mice were administered a high-fat diet plus ethanol suggested that an estrogen conjugate might benefit both NAFLD and AFLD patients [42]. However, reports showing the expression patterns of estrogen-receptor α in human hepatic nuclei are rare [43].

Our data indicates that both pERK in hepatic stellate cells and estrogen-receptor α in hepatocytes may be linked to the development of hepatic steatosis. pERK was expressed in hepatic stellate cells in all hepatic steatosis cases examined. Previous studies of rodent liver tissue also implicate pERK in the development of hepatic steatosis, as well as steatohepatitis [44–46]. Moreover, pERK signaling in activated hepatic stellate cells was pro-fibrogenic in NAFLD [15] and AFLD [47] patients in previous studies. Estrogen-receptor α was sufficiently expressed in all NAFLD and AFLD cases. In chicken primary hepatocytes, estrogen enhanced hepatic fatty acid synthesis [48]. In NAFLD patients with elevated serum estrogen levels [11], estrogen entered hepatocytes, resulting in the translocation of ligand-bound estrogen-receptors to the nucleus [49]. Serum estrogen levels are known to be elevated in alcoholic men [50], perhaps owing, at least in part, to the presence of biologically active phytoestrogen (derived from plant-based ingredients such as grains, fruits, and hops) in alcoholic beverages [51].

To our knowledge, this is the first report of mTOR nuclear expression in human liver tissue. mTOR was previously believed to localize exclusively to the cytoplasm; however, recent reports document its presence in multiple intracellular compartments, including the nucleus, as well as shuttling between the cytoplasm and nucleus. However, a specific nuclear function for mTOR has yet to be established [51–53].

This retrospective study does have some limitations. First, many of the NAFLD patients were young men at less-advanced disease stage. This reflects the inclusion of men who underwent medical check-ups prior to mandatory military service in Korea; our hospital is among the institutions providing official health documentation. Second, it was not determined whether mTOR, STAT3 and ERK belong to leptin signaling pathways. Leptin and leptin-receptor antibodies used in this study did not specifically recognize their target antigens in human liver tissue. This occurred despite previous use of these antibodies to specifically detect leptin and leptin-receptor in the hepatocellular carcinoma (Additional file 2), breast, biliary tract, appendix and stomach [54, 55].

## Conclusions

Here we have shown that pSTAT3 expression correlates with severe histomorphologic features in hepatic steatosis, and suggest that diffuse expression of estrogen-receptor α may lessen severity. Hence, both proteins may play a role in mechanisms of disease progression in patients with hepatic steatosis. Our results also implicate pERK and estrogen-receptor α in the development of hepatic steatosis. How these proteins modulate the disease development process is, however, unclear and warrants further study.

## Additional files

Additional file 1: Differential features between non-alcoholic fatty liver disease (NAFLD) subgroups stratified by NAS and SAF. (DOCX 47 kb)

Additional file 2: Immunohistochemical staining for leptin (A-20, 1:50; Santa Cruz Biotechnology, Santa Cruz, CA, USA) and leptin-receptor (B-3, 1:25; Santa Cruz Biotechnology) in hepatic steatosis cases, and non-neoplastic hepatocytes and carcinoma cells from hepatocellular carcinomas. (**a-b)** Hepatic steatosis with nuclear and/or cytoplasmic staining of leptin (**a**), and diffuse granular staining of leptin-receptor (**b**). (**c-e**) Leptin in hepatocellular carcinomas. Nuclear and cytoplasmic staining of leptin in non-neoplastic hepatocytes (left side of each picture), and negative (**c**), weak (**d**) or strong (**e**) leptin staining in hepatocellular carcinoma cells (right side of each picture). (**f-g**) Leptin-receptor in hepatocellular carcinomas. Diffuse granular cytoplasmic expression of leptin-receptor in non-neoplastic hepatocytes (left), and weak focal (**f**) or strong diffuse (**g**) staining of leptin-receptor in hepatocellular carcinoma cells (right). (JPEG 4962 kb)

## Abbreviations

AFLD: Alcoholic fatty liver disease
ALT: Alanine transaminase
BMI: Body mass index
mTOR: Mammalian target of rapamycin
NAFLD: Non-alcoholic fatty liver disease
NASH CRN NAS: Non-alcoholic Steatohepatitis Clinical Research Network NAFLD activity score
pERK: Phosphorylated extracellular signal-regulated kinase
pSTAT: Phosphorylated signal transducer and activator of transcription
SAF: Steatosis, activity, and fibrosis
