# Supplementary material for: Expression patterns of STAT3, ERK and estrogen-receptor α are associated with development and histologic severity of hepatic steatosis: a retrospective study
Source: Diagn Pathol. 2018 Apr 3;13:23. doi: 10.1186/s13000-018-0698-8 (PMC5883355; doi:10.1186/s13000-018-0698-8)
Supplement: Supplementary file 1 — Differential features between non-alcoholic fatty liver disease (NAFLD) subgroups stratified by NAS and SAF. (DOCX 47 kb) [file 13000_2018_698_MOESM1_ESM.docx]

Additional File 1. Differential features between non-alcoholic fatty liver disease (NAFLD) subgroups stratified by NAS and SAF

|  | NAFLD Activity Score (NAS) | |  | Steatosis Activity Fibrosis  (SAF) | |
| --- | --- | --- | --- | --- | --- |
|  | Severe NAFLD | Mild NAFLD |  | Significant NAFLD | Mild NAFLD |
|  | (NAS ≥ 5) | (NAS 1~4) |  | (A ≥2 &/or F≥2) | (A < 2 & F <2) |
|  | (n = 5) | (n = 13) |  | (n = 11) | (n = 7) |
| Steatosis (score) |  |  |  |  |  |
| 5-33% (1) | 0 | 4 (31%) |  | 2 (18%) | 2 (29%) |
| >33-66% (2) | 1 (20%) | 4 (31%) |  | 4 (36%) | 1 (14%) |
| >66% (3) | 4 (80%) | 5 (38%) |  | 5 (45%) | 4 (57%) |
| Lobular inflammation (inflammatory foci/200x field) (score) | | | | | ***** |
| <2 (1) | 3 (60%) | 12(92%) |  | 8 (73%) | 7 (100%) |
| 2-4 (2) | 2 (40%) | 1 (8%) |  | 3 (27%) | 0 |
| Ballooning degeneration (score) | | ***** |  |  | ***** |
| none (0) | 1 (20%) | 9 (69%) |  | 3 (27%) | 7 (100%) |
| few (1) | 3 (60%) | 4 (31%) |  | 7 (64%) | 0 |
| many (2) | 1 (20%) | 0 |  | 1 (1%) | 0 |
| Fibrosis |  |  |  |  |  |
| absent | 1 (20%) | 9 (69%) |  | 5 (45%) | 5 (71%) |
| present | 4 (80%) | 4 (31%) |  | 6 (55%) | 2 (29%) |
| mTOR in hepatocytes | | |  |  |  |
| negative | 1 (20%) | 4 (31%) |  | 2 (18%) | 3 (43%) |
| positive | 4 (80%) | 9 (69%) |  | 9 (82%) | 4 (57%) |
| pSTAT3 in hepatocytes | | |  |  |  |
| negative | 3 (60%) | 12 (92%) |  | 8 (73%) | 7 (100%) |
| positive | 2 (40%) | 1 (8%) |  | 3 (27%) | 0 |
| pERK in hepatocytes | | |  |  |  |
| negative | 5 (100%) | 9 (69%) |  | 8 (73%) | 6 (86%) |
| positive | 0 | 4 (31%) |  | 3 (27%) | 1 (14%) |
| Estrogen-receptor α in hepatocytes | | |  |  |  |
| focal | 0 | 0 |  | 0 | 0 |
| diffuse | 5 (100%) | 13 (100%) |  | 11 (100%) | 7 (100%) |
| pERK in hepatic stellate cells | |  |  |  |  |
| focal | 2 (40%) | 6 (46%) |  | 4 (36%) | 4 (57%) |
| diffuse | 3 (60%) | 7 (54%) |  | 7 (64%) | 3 (43%) |

******P* < 0.05
