# Supplementary figures and images for: Expression patterns of STAT3, ERK and estrogen-receptor α are associated with development and histologic severity of hepatic steatosis: a retrospective study
Source: Diagn Pathol. 2018 Apr 3;13:23. doi: 10.1186/s13000-018-0698-8 (PMC5883355; doi:10.1186/s13000-018-0698-8)

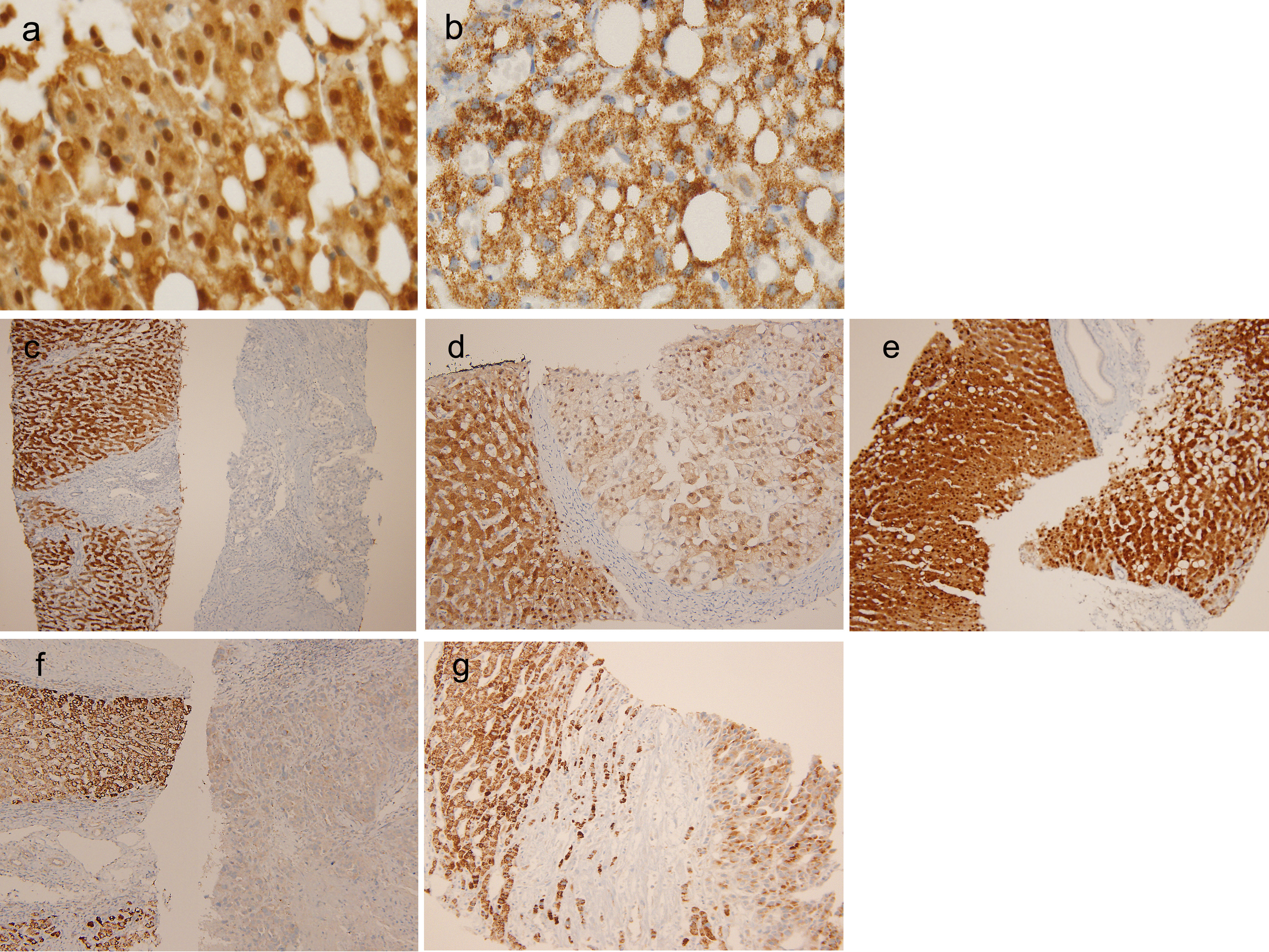

Supplement: Supplementary file 2 — Immunohistochemical staining for leptin (A-20, 1:50; Santa Cruz Biotechnology, Santa Cruz, CA, USA) and leptin-receptor (B-3, 1:25; Santa Cruz Biotechnology) in hepatic steatosis cases, and non-neoplastic hepatocytes and carcinoma cells from hepatocellular carcinomas. (a-b) Hepatic steatosis with nuclear and/or cytoplasmic staining of leptin (a), and diffuse granular staining of leptin-receptor (b). (c-e) Leptin in hepatocellular carcinomas. Nuclear and cytoplasmic staining of leptin in non-neoplastic hepatocytes (left side of each picture), and negative (c), weak (d) or strong (e) leptin staining in hepatocellular carcinoma cells (right side of each picture). (f-g) Leptin-receptor in hepatocellular carcinomas. Diffuse granular cytoplasmic expression of leptin-receptor in non-neoplastic hepatocytes (left), and weak focal (f) or strong diffuse (g) staining of leptin-receptor in hepatocellular carcinoma cells (right). (JPEG 4962 kb) [file 13000_2018_698_MOESM2_ESM.jpg]
